# Understanding implementability in clinical trials: a pragmatic review and concept map

**DOI:** 10.1186/s13063-021-05185-w

**Published:** 2021-03-26

**Authors:** Miranda S. Cumpston, Steven A. Webb, Philippa Middleton, Greg Sharplin, Sally Green, for the Australian Clinical Trials Alliance Reference Group on Impact and Implementation of CTN Trials, Karen Best, Frank Bloomfield, Alan Cass, Paul Cohen, Sue Crengle, Louise Cullen, Dashiell Gantner, Heide Gaulke, Davina Ghersi, Sally Green, Paul Glasziou, Tiffany Harris-Brown, Stephen Jan, David Johnson, Samantha Keogh, Chris Levi, Philippa Middleton, Sandra Peake, Angela Scheppokat, Paul Scuffham, Greg Sharplin, Tim Shaw, David Story, Helena Teede, Val Theisz, Judith Trotman, Steve Webb, Carolina Weller, Anne Woollett, Sophia Zoungas

**Affiliations:** 1 School of Public Health and Preventive Medicine, Monash University, 553 St Kilda Rd, Melbourne, VIC 3004 Australia; 2 South Australian Health & Medical Research Institute, PO BOX 11060, Adelaide, SA 5001 Australia; 3 Rosemary Bryant AO Research Centre, Clinical and Health Sciences Unit, University of South Australia, City East Campus, Playford Building P4-27F, North Terrace, Adelaide, SA 5000 Australia

**Keywords:** Clinical trials, Late-phase trials, Implementability, Pragmatic trials, Applicability, Implementation, Knowledge translation

## Abstract

**Background:**

The translation of evidence from clinical trials into practice is complex. One approach to facilitating this translation is to consider the ‘implementability’ of trials as they are designed and conducted. Implementability of trials refers to characteristics of the design, execution and reporting of a late-phase clinical trial that can influence the capacity for the evidence generated by that trial to be implemented. On behalf of the Australian Clinical Trials Alliance (ACTA), the national peak body representing networks of clinician researchers conducting investigator-initiated clinical trials, we conducted a pragmatic literature review to develop a concept map of implementability.

**Methods:**

Documents were included in the review if they related to the design, conduct and reporting of late-phase clinical trials; described factors that increased or decreased the capacity of trials to be implemented; and were published after 2009 in English. Eligible documents included systematic reviews, guidance documents, tools or primary studies (if other designs were not available). With an expert reference group, we developed a preliminary concept map and conducted a snowballing search based on known relevant papers and websites of key organisations in May 2019.

**Results:**

Sixty-five resources were included. A final map of 38 concepts was developed covering the domains of validity, relevance and usability across the design, conduct and reporting of a trial. The concepts drew on literature relating to implementation science, consumer engagement, pragmatic trials, reporting, research waste and other fields. No single resource addressed more than ten of the 38 concepts in the map.

**Conclusions:**

The concept map provides trialists with a tool to think through a range of areas in which practical action could enhance the implementability of their trials. Future work could validate the strength of the associations between the concepts identified and implementability of trials and investigate the effectiveness of steps to address each concept. ACTA will use this concept map to develop guidance for trialists in Australia.

**Trial registration:**

This review did not include health-related outcomes and was therefore not eligible for registration in the PROSPERO register.

## Background

Clinical trialists conduct trials with the aim of improving health care and health outcomes for the community. Similarly, research funders wish to ensure the trials they fund represent good value for money [1, 2], including the appropriate use of public or charitable funds, and increasingly seek to demonstrate their impact (or capacity for impact) [3–5]. Nevertheless, much has been written on delays between research and implementation [6], and the extent to which research does not meet the needs of users and may be wasted [2, 7].

In this study, we focus on the concept of *implementability* of a trial’s findings. We define implementability as the characteristics of the design, execution and reporting of a clinical trial, typically a late-phase trial, that influence the capacity for the evidence generated by that trial to be implemented. In other words, the characteristics that mean the findings of a trial *could* be implemented, should that be appropriate.

We distinguish this concept from the concept of *implementation*. A decision about whether and how an intervention *should* be implemented must wait until the results of the trial are known and will depend on a range of contextual factors.

**Fig. 1:**
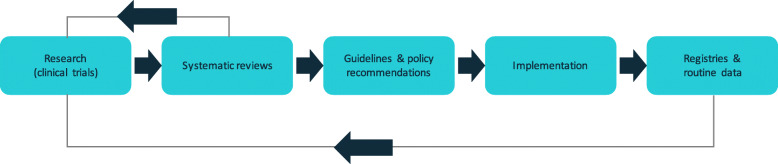
Evidence and implementation cycle. Adapted from: *Australia’s next generation evidence ecosystem: Maximising the value of research for better health*, Federal Budget Submission 2018–2019, Cochrane Australia; 2018

The relationship between the conduct of a clinical trial and the subsequent implementation of the knowledge it generates into policy and practice is complex, even for late-phase trials testing interventions that are under consideration for implementation. Any single trial exists in the context of a field of research and contributes to cycles of implementation, learning and further research (see Fig. 1). There may be multiple stages of activity required before policy or clinical practice could, or should, be expected to change as the result of a trial, including synthesis of the trial’s findings in a systematic review, development of a clinical practice guideline, funding and policy decisions, integration into clinical care settings, dissemination to clinicians and shared decision-making with consumers. The research field of implementation science has contributed much to our understanding of these complexities and continues to explore the systemic and personal factors that can influence the effectiveness of knowledge translation interventions and the motivations and actions of individual stakeholders [7, 8].

Although much of this work falls outside the scope of triallists, there are steps that can be taken during the design, conduct and reporting of trials to enhance the capacity for trials to be useful for, and used by, those involved in implementation activities. By considering the needs of the end users of trial findings—including clinicians, policy makers, health service managers, patients, knowledge brokers, guideline developers and systematic review authors—can the implementability of trials be enhanced, and barriers to possible future implementation removed?

While the concept of implementability has been applied recently to clinical practice guidelines, given their intended use for implementation of clinical practice change [9–14], we were not aware of any previous published work applying this concept to clinical trials.

The Australian Clinical Trials Alliance (ACTA) is a peak body representing Clinical Trials Networks (CTNs), Coordinating Centres and Clinical Quality Registries in Australia [15]. ACTA’s members primarily support the conduct of investigator-initiated, multi-site clinical trials across a variety of clinical specialties. Most focus their activities in Australia; some operate routinely across Australia, New Zealand and the South-East Asian region; and some also lead international multi-site trials or support the Australian sites of international, multi-site trials.

ACTA is working to develop practical guidance to support CTNs and Centres conducting clinical trials, including guidance to enhance the implementability of trials. To inform this guidance, we conducted a pragmatic literature review to develop a concept map of actions in the design, conduct and reporting of clinical trials that may enhance the implementability of these trials.

### Objectives

The objective of the review was to map features of the design, conduct and reporting of clinical trials that promote implementability, to inform the development of guidance for Australian Clinical Trials Networks and Coordinating Centres on how to enhance implementability of trials.

## Methods

The review was designed in consultation with an existing expert reference group including experienced trialists and implementation scientists, established to provide ongoing advice to ACTA. A protocol was developed in advance and approved by the reference group prior to commencement of the search. The protocol is available from the authors on request.

Due to funding and time constraints, this review was conducted using a pragmatic approach to inform the development of guidance and is not a systematic review.

This review did not include health-related outcomes and was therefore not eligible for registration in the PROSPERO register. A PRISMA reporting guideline checklist for this paper is provided in Additional file 1.

### Preliminary concept framework

**Fig. 2:**
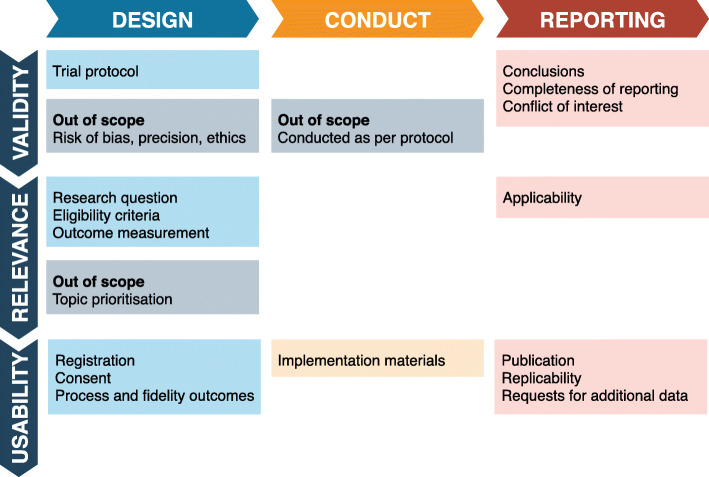
Preliminary concept framework. Broad concepts expected to be relevant to implementability at each stage of the trial process

We developed a preliminary framework to identify concepts expected to be relevant to implementability at each stage of the trial process and to determine which areas should be considered out of scope for this study (see Fig. 2). The framework was drafted by one author (MC) and refined by consensus with the remaining authors and the expert reference group. To organise and understand the concepts, we categorised them into domains according to trial stage (design, conduct of the trial or reporting), and broad subject categories (concepts relevant to the validity of the trial, its relevance to end users and the ease of use of the trial findings).

The authors then populated the framework with specific candidate concepts in as much detail as possible, in consultation with the expert reference group. We aimed for broad inclusivity to encourage consideration of implementability from diverse perspectives and inform the development of an inclusive search strategy. Two authors (MC, SW) edited the preliminary list to remove duplicate or out-of-scope concepts and ensure clarity of wording. Thirty-three candidate concepts were mapped across the nine domains of the framework. It was intended that this concept map would be iteratively added to and amended in accordance with the review’s findings. The complete preliminary concept map is provided in Section A, Additional file 2.

### Eligibility criteria

Documents were included in the review if they met all of the following criteria:

1. *Described features of late-stage clinical trials.*
  Late-stage trials were defined as trials intended to inform decisions about whether the candidate intervention should be adopted into practice or policy, should the results prove definitive. This may include features of other research types (such as systematic reviews) that could be applied to late-stage clinical trials. Early-phase trials and mechanistic trials, intended to learn more about the potential value of a candidate intervention, the processes underpinning it and/or whether further trials are warranted, were considered out of scope and excluded.
2. *Described factors relating to the design, conduct and reporting of trials.*
  This included any activity from the decision to conduct a trial to its final reporting. Issues relating to topic prioritisation and identifying important research questions prior to the planning of individual trials, and interventions to achieve implementation separate or subsequent to the completion of trials, were considered out of scope and so excluded (see Fig. 2).
3. *Described factors that were perceived or demonstrated to change the capacity of clinical trials to be used in implementation into policy or clinical practice.*
  This included aspects of relevance and applicability, accessibility by end users (including clinicians, policy makers, managers, consumers, implementation scientists and guideline developers), usability by end users and any other concepts identified. Issues relating to minimising risk of bias and issues relating to statistical power and precision in trials were considered out of scope and so excluded (see Fig. 2).
4. *Were of an eligible type, including guidance or policy documents based on evidence-informed processes, systematic reviews of relevant primary studies, and tools developed using evidence or consensus methods.*
  Primary studies were only included where the above designs were not available. Relevant primary studies could include studies of trial methodology (such as surveys of methodology and reporting of published trials) or of trial implementation (such as qualitative studies of barriers and facilitators to implementation, or surveys of trial uptake for implementation). Reports of individual clinical trials were not eligible.
5. Were in English.

Where multiple documents addressing similar concepts were identified (for example, more than one empirical study documenting a particular practice in reports of published trials), a hierarchical approach was taken: studies looking at international practice and across all areas of healthcare were preferred over studies in single countries or specific areas of practice. Documents were also excluded if they had been superseded by a more recent or detailed document (for example, if an organisation replaced a brief policy document with more detailed guidance, or if a systematic review had been updated).

One final criterion for exclusion was added retrospectively, during the screening process, on the advice of the reference group. Documents were excluded if they had been published more than 10 years ago (i.e. before 2009), as we felt that older documents may not reflect the current theoretical understanding of implementation or current practice in trials.

### Search strategy

The preliminary concept map was used to inform the selection of search terms and topics, to ensure that diverse relevant concepts were captured regardless of the focus or language used in candidate records. The following search activities were completed as at 20 May 2019 by one author (MC):

1. A list of key known documents was generated and confirmed with all authors and the expert reference group, including published literature and guidance documents (see Section B, Additional file 2).
2. Snowballing was conducted from these known documents using reference lists, linked publications in series or relating to the same tool, and the ‘similar articles’ function in PubMed [16] (for those documents indexed in PubMed).
3. A list of key tools and checklists likely to be relevant to implementability was generated (see Section C, Additional file 2).
4. A targeted search of websites of key organisations was conducted using the navigation menus of each site and the search term ‘implementation’, as well as additional terms depending on the language and focus areas of each organisation, including ‘pragmatic’, ‘comparative effectiveness’ and ‘stakeholder’. The organisations searched were:
  Agency for Healthcare Research and Quality (AHRQ), USA [17]
  Canadian Institutes of Health Research (CIHR) [18]
  Clinical Trials Transformation Initiative (CTTI), USA [19]
  Cochrane Library [20]
  COMET (Core Outcome Measures in Effectiveness Trials) Initiative [21]
  EQUATOR (Enhancing the QUAlity and Transparency Of health Research) Network [22]
  Guidelines International Network (G-I-N) [23]
  INVOLVE, UK [24]
  Joanna Briggs Institute (JBI) [25]
  Multi-Regional Clinical Trials (MRCT) Center of Brigham and Women’s Hospital and Harvard, USA [26]
  National Collaborating Centre on Methods and Tools (NCCMT), Canada [27]
  National Health and Medical Research Council (NHMRC), Australia [28]
  National Institutes of Health (NIH), USA [29]
  National Institute for Health Research (NIHR), UK [30]
  Patient-Centered Outcomes Research Institute (PCORI), USA [31]
  REWARD Alliance [32]
5. Any additional documents suggested by the expert reference group during the course of the review were added.

### Selection

The search results were screened at the level of title and abstract. The full text of potentially included documents was then retrieved. Final decisions about the relevance of documents were made on the basis of the full text. Screening and eligibility decisions were made by one author (MC) using Endnote. Where any decision was unclear, a second author was consulted (SG) and consensus reached.

### Data collection

Data were extracted by one author (MC) using Microsoft Excel, including:

- Concepts identified that are relevant to clinical trials and may increase or decrease the implementability of clinical trials
- Descriptions of research design, conduct and reporting that would enhance these concepts/factors
- Descriptions of guidance/tools to enable clinical trials to include features shown to enhance implementability in their trial design and conduct
- The organisation or authors who developed or endorsed the document, the country/location (if applicable) and the date of publication

Critical appraisal of the included documents was not performed, as the objective of the review was to map the existing information.

### Analysis

Concepts relevant to implementability and resources or guidance relevant to each concept were mapped against the initially developed concept map. Gaps in the availability of resources and guidance were noted. The concept map was amended iteratively by adding concepts identified through the literature search, and removing concepts not confirmed by the literature as important to implementability. Given the number of concepts involved, it was not feasible to present detailed definitions or descriptions of each concept, but the number, type and references of source documents supporting each concept were tabulated.

## Results

### Results of the search

**Fig. 3:**
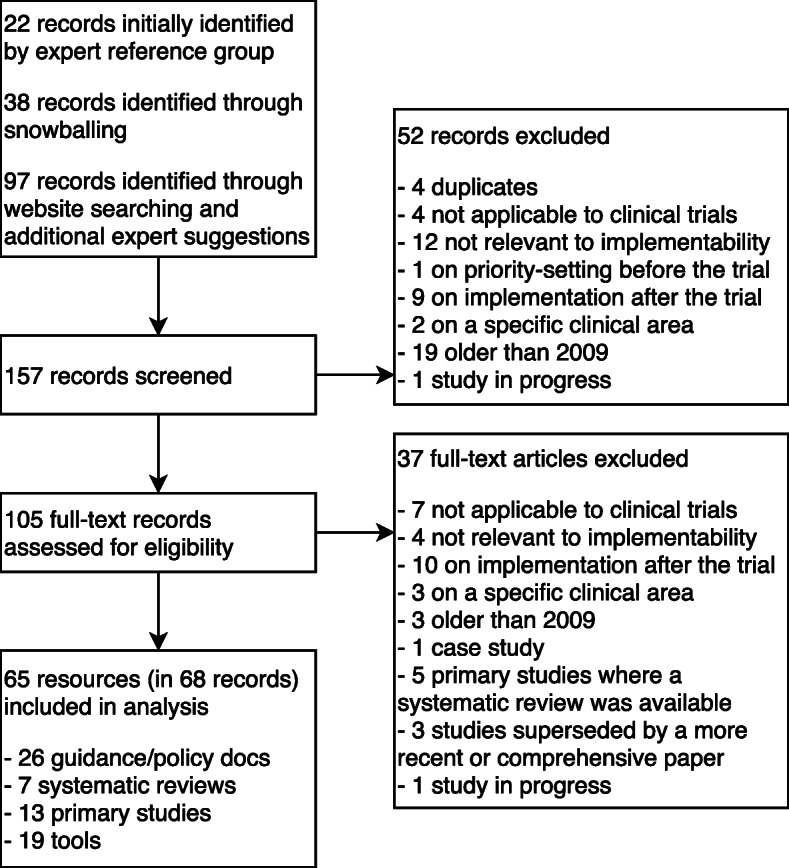
PRISMA flow diagram of documents identified in the search

The results of the search and the reasons for exclusion are outlined in Fig. 3.

An initial list of 22 records of documents was identified in collaboration with the expert reference group (see Sections B and C, Additional file 2). An additional 38 records were identified through snowballing, and 97 were identified through website searching and additional expert suggestions, making a total of 157 potentially relevant records.

Fifty-two records of documents were excluded at the title and abstract stage, and the full text was retrieved for the remaining 105 records. An additional 37 records were excluded on the basis of their full text, leaving 68 included records describing 65 resources (some resources had more than one report), including descriptive studies, tools and guidance documents. A full list of excluded records is presented in Section D, Additional file 2.

### Description of included resources

A detailed table of characteristics of included resources is presented in Additional file 3.

Of the 65 included resources:

- Twenty-six were guidance documents, of which 11 were produced by an institution or organisation and 15 were produced by individuals or groups of experts.
- Four were studies (two systematic and two other literature reviews) documenting a practice in trials that enhanced implementability, such as the impact of patient engagement on research or facilitators to the uptake of evidence into policy.
- Sixteen were studies (six systematic reviews, one of which also included information in the previous category of benefit or good practice, and 11 cross-sectional studies) documenting a problem or less-than-optimal practice in trials, such as discordance between the protocol and the published trial, discordance in interpretation of trials, incomplete reporting, applicability and consent for data re-use.
- Nineteen were practical tools or checklists (one was a repository of multiple tools) designed to assist either trialists or those interpreting or using trial results to report or assess specific aspects.

Most of the studies were internationally applicable, although some had a specific geographic scope, such as guidance produced by funding organisations (such as NIHR in the UK, CIHR in Canada or NHMRC in Australia), or descriptive studies of research conducted in a specific country or region.

### Findings

**Fig. 4:**
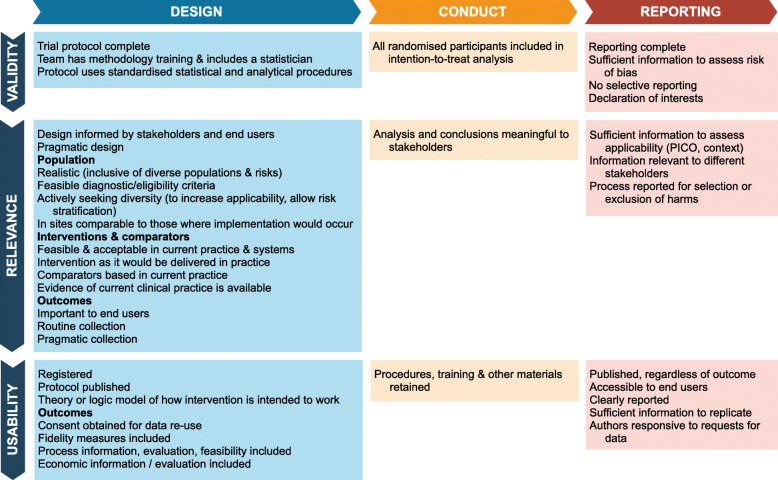
Final map of concepts enhancing implementability in clinical trials

The final concept map includes 38 items and is presented in Fig. 4. A list of items added to and removed from the initial concept map is provided in Table 1. Twenty-nine of the 33 initially proposed concepts were supported by the literature as being related to the implementability of trials. Four preliminary concepts were removed from the final concept map. We note that these concepts are likely to remain relevant for the good conduct of clinical trials, but may sit outside the domain of implementability. Ten additional concepts were identified and added to the concept map, including one (interventions acceptable in current practices and systems) that was merged with an existing concept of feasibility as the two concepts were discussed together in the literature.

**Table 1:** Changes made to initial concept map

Items removed from the initial concept map	Items added to the initial concept map
Validity: Design • Trial protocol considers implementability Relevance: Design • Outcomes are persuasive (prespecified outcomes would be sufficient to motivate change) • Evidence of persuasiveness of outcomes is available Validity: Reporting • Conclusions supported by data	Validity: Design • Team has methodology training and includes a statistician • Protocol uses standardised statistical and analytical procedures Relevance: Design • Pragmatic design • Interventions acceptable in current practices and systems Usability: Design • Theory or logic model specified for how intervention is intended to work Validity: Conduct • All randomised participants included in the intention-to-treat analysis Relevance: Conduct • Analysis and conclusions meaningful to stakeholders Relevance: Reporting • Information relevant to different stakeholders • Process reported for selection or exclusion of harms Usability: Reporting • Clearly reported

Much of the focus at the design stage of a trial was on ensuring that the trial would answer research questions of importance to consumers and other end users of the findings. Strategies to achieve this included consultation and involvement of stakeholders, as well as ensuring that diverse affected populations are included in trials. Another frequent focus was to enhance the feasibility of implementing the trial’s protocols in routine clinical practice, including pragmatic interventions that are feasible and acceptable in current practice, and using data for outcome measurement that is already routinely collected, or at minimum as pragmatically collected as possible.

In reporting the trial, the concepts identified focused on clarity and trust. While the risk of bias in the methods used in trials was considered out of scope, the reporting of trial methods can be a barrier to trust and implementation if readers are unable to judge the risk of bias for themselves. Similar concepts applied to judgements by end users about the applicability of trial findings to different populations or contexts. Even the selection of statistical and other analysis methods at the design phase can affect the capacity of end users to interpret and compare findings between clinical trials. The use of skilled methodologists and comparable or standardised analysis methods could alleviate this.

Individual concepts were supported by between one and 17 resources. Five concepts were supported by more than ten resources each. Two of these concepts related to trial design: 15 resources indicated that design should be informed by stakeholders and end users and 13 resources indicated that outcomes should be important to end users. Three of these concepts related to trial reporting: 17 resources indicated that sufficient information should be reported to assess applicability; 13 indicated that sufficient information should be reported to replicate the intervention; and 12 indicated that sufficient information should be reported to assess the risk of bias in the trial. Complete information on which included documents supported each concept, including the resource type (guidance, descriptive information and tools), is presented in Additional file 4. Each concept in the map is briefly stated, but trialists considering adopting any of the listed measures should consult the original resources for a fuller understanding of their meaning and implications.

The individual included resources referenced between one and ten of the 38 concepts included in the final concept map, and only eleven of the 68 included resources referenced five or more concepts. It was common for included resources to focus on a particular aspect of implementability, such as consumer engagement, knowledge translation for policy, reporting or pragmatic trials. Only two included resources appeared to target the concept of implementability as a whole, more or less consistently with the concept as defined in our study: Resource IDs 34 and 45 (see Additional files 3 and 4), both expert guidance papers. These two papers supported the largest number of concepts (ten and nine, respectively) and contributed three and two (respectively) new concepts to the map not identified at the preliminary stage.

## Discussion

This study explored aspects of implementability in the design, conduct and reporting of clinical trials. In reviewing the available literature, we have developed a structured framework of actions that trialists can take in the design, conduct and reporting of trials to better enable the implementation of their findings into practice and policy. We mapped 38 concepts contributing to implementability, drawing on 68 resources. Drawing on a range of guidance documents, empirical studies of trial design and factors influencing implementation, and practical tools for trialists, our findings indicate that while some of these concepts are well covered in the literature, no previous work has drawn together these concepts and mapped the construct of implementability for trials in this overarching way. Existing resources on implementability incorporated no more than ten of our included concepts, and frequently fewer, each being likely to focus on more specific aspects of implementability. In bringing these concepts together, our map could form the foundation of a more comprehensive approach to enhancing the implementability of trials.

Some concepts added to the map through our literature review were not initially identified by the expert working group. These included the need for the trial team to have methodology training and include a statistician and the use of standardised statistical and analytical procedures. While potentially more relevant to reducing the risk of bias of a trial, and so out of scope for this review, these concepts were discussed by the listed resources as additionally important to implementability, considering aspects such as the appropriateness and consistency of analysis approached between studies. For this reason, we included them in the map.

For practical application by trialists, it may not be feasible for any single trial to address all 38 of the concepts included in the map. Although the concepts in this map are broadly applicable across late-phase trials in any clinical area, some concepts are contingent on, or may conflict with, the nature of the research question. For example, very novel interventions might be less straightforward to integrate into existing care settings in the manner of a pragmatic trial, and some outcomes important to stakeholders may not be included in routine data collection. Other concepts within the map are always feasible and do not require additional resources to implement, aside from the relevant skills and knowledge within the trial team, such as providing more detailed reporting of both the trial methodology and the interventions assessed. Trialists will be best placed to judge which of the concepts outlined in this map might be applicable in practice for any individual trial.

We note that most efforts to enhance implementability must take place prior to and separately from the decision whether or not to implement the findings of a given trial. Some trials may be inappropriate to implement into practice and policy due to the nature of their findings, for example trials whose findings indicate that novel interventions are ineffective, or trials of interventions that are not feasible in a specific context. However, efforts to improve the implementability of trials may remove additional barriers to appropriate implementation and enhance the usefulness of the trial’s findings to clinical and policy decision-makers.

Limitations of this study include the snowballing approach taken to identifying the literature, and directing our search through the lens of a preliminary concept map may have limited our findings. However, we endeavoured to use the preliminary concept map and consultation with our expert reference group to expand rather than limit the concepts included in our search. It is likely that a more systematic search would have identified additional relevant resources, and it is possible that these resources may have contributed additional concepts for our map. Double-screening of included resources by two authors may also have identified additional resources, or altered their interpretation within the concept map.

Further work will be important to develop the concepts outlined by this pragmatic review. Future work could include documenting case studies or exemplars of implementable trials, additional studies using alternative methods to add to or validate the concepts in the map, or further investigation of the relative importance to end users of specific concepts, particularly those with fewer supporting documents.

It was noteworthy that a minority of the included studies included empirical evidence documenting either a problem or the effectiveness of good practice relating to implementability. The bulk of resources in this area were based on expert opinion. Empirical research would be valuable to investigate how trialists could integrate these concepts into the design, conduct and reporting of individual trials. In particular, evaluation of the effects of actions that are likely to be more resource-intensive or complex to implement, such as consultation, integration with practice, diverse recruitment and process evaluation, would be important to support their more widespread uptake.

In the short term, this study’s findings will contribute to the development of practical guidance by ACTA for use by Australian trialists.

## Conclusions

This study presents a detailed map identifying 38 concepts that can enhance the implementability of clinical trials—that is, their capacity to be implemented in policy or practice, should their findings be appropriate for implementation. This concept map can now be used by trialists to think through a range of areas in which practical action could enhance the implementability of their trials and to identify available guidance and resources to inform their decision-making in the design, conduct and reporting of clinical trials. Future work could validate the strength of the associations between the concepts identified and implementability of trials and investigate the effectiveness of steps to address each concept. ACTA will use this concept map to inform the development of further practical guidance.

## Supplementary Information

**Additional file 1.** PRISMA checklist. Completed checklist for reporting of reviews.

**Additional file 2.** Preliminary concept map, reference lists and excluded studies. Additional information relating to the search for included resources, including preliminary list of key papers used for snowballing, preliminary list of relevant tools and table of excluded resources.

**Additional file 3.** Characteristics of included resources. A complete list of included resources, including year of publication, resource type, design, producing organisation, geographic scope, description and full reference information.

**Additional file 4: Figure.** Supporting resources for each concept. A more detailed version of the concept map displaying the specific supporting references for each concept.

## Data Availability

Data generated and analysed during this study are included in this published article and its supplementary information files. The study protocol is available from the corresponding author on request.

## Abbreviations

ACTA: Australian Clinical Trials Alliance
CTN: Clinical Trials Network
PROSPERO: International prospective register of systematic reviews
