# Supplementary material for: Understanding implementability in clinical trials: a pragmatic review and concept map
Source: Trials. 2021 Mar 26;22:232. doi: 10.1186/s13063-021-05185-w (PMC7995762; doi:10.1186/s13063-021-05185-w)
Supplement: Supplementary file 2 — Additional file 2. Preliminary concept map, reference lists and excluded studies. Additional information relating to the search for included resources, including preliminary list of key papers used for snowballing, preliminary list of relevant tools and table of excluded resources. [file 13063_2021_5185_MOESM2_ESM.pdf]

Additional File 2: Preliminary reference lists and excluded studies

Understanding implementability in clinical trials: a pragmatic review and concept map

Miranda Cumpston, Steve Webb, Philippa Middleton, Greg Sharplin, Sally Green for the Australian Clinical Trials Alliance Reference Group on Impact and Implementation of CTN Trials.

A. Preliminary concept map

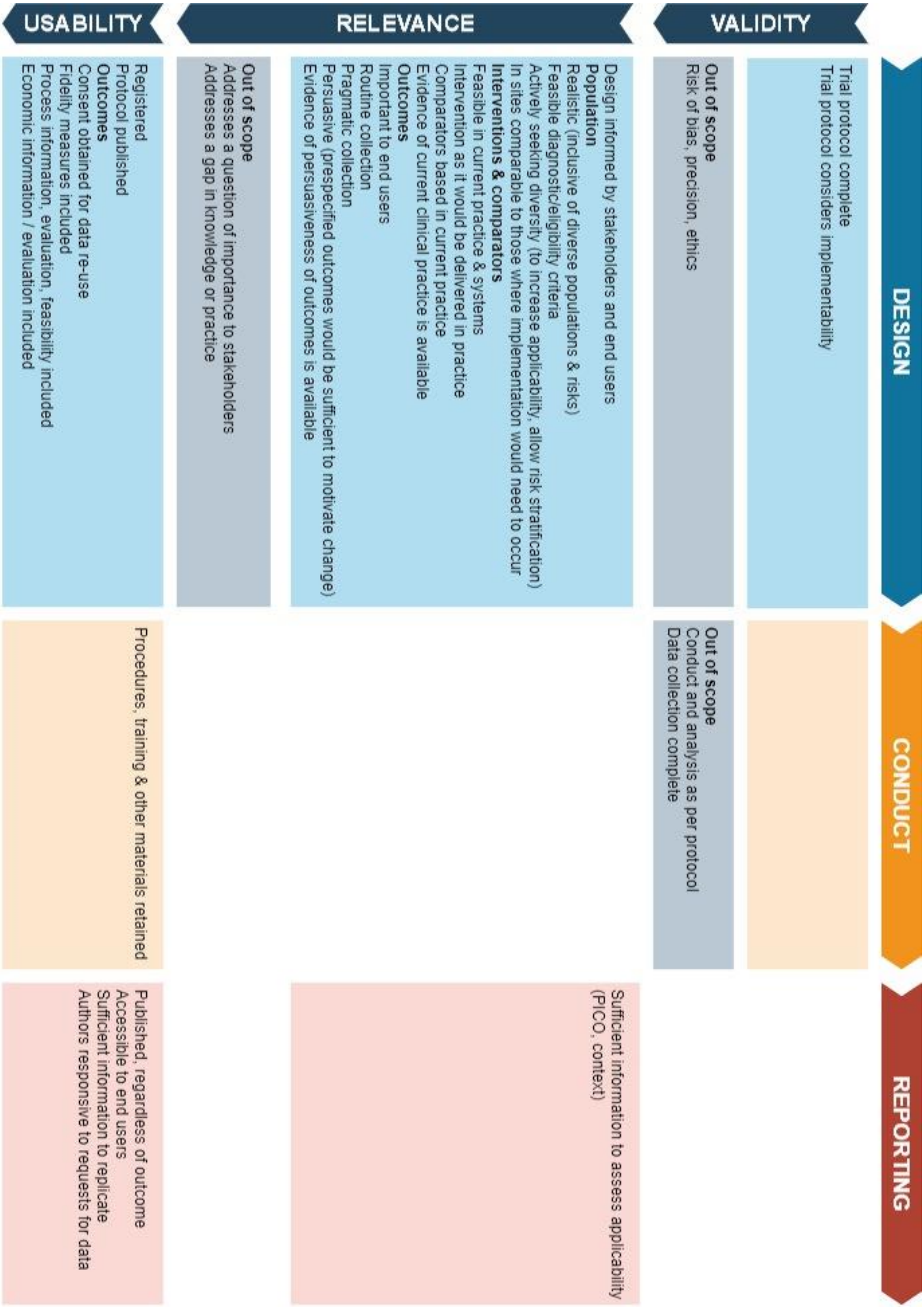

B. Preliminary list of key papers used for snowballing

Brennan, S. E., M. Cumpston, M. L. Misso, S. McDonald, M. J. Murphy and S. E. Green (2016). "Design and formative evaluation of the Policy Liaison Initiative: a long-term knowledge translation strategy to encourage and support the use of Cochrane systematic reviews for informing health policy." Evidence & Policy: A Journal of Research, Debate and Practice 12(1): 25-52.

Campbell, S., S. Benita, E. Coates, P. Davies and G. Penn (2007). Analysis for policy: evidence-based policy in practice. London, HM Treasury.

Cook, J. M., C. O'Donnell, S. Dinnen, J. C. Coyne, J. I. Ruzek and P. P. Schnurr (2012). "Measurement of a model of implementation for health care: toward a testable theory." Implementation Science 7(1): 59.

Damschroder, L. J., D. C. Aron, R. E. Keith, S. R. Kirsh, J. A. Alexander and J. C. Lowery (2009). "Fostering implementation of health services research findings into practice: a consolidated framework for advancing implementation science." Implementation Science 4(1): 50.

Dechartres, A., L. Trinquart, I. Atal, D. Moher, K. Dickersin, I. Boutron, E. Perrodeau, D. G. Altman and P. Ravaud (2017). "Evolution of poor reporting and inadequate methods over time in 20 920 randomised controlled trials included in Cochrane reviews: research on research study." BMJ 357: j2490.

Edinger, T. and A. M. Cohen (2013). "A large-scale analysis of the reasons given for excluding articles that are retrieved by literature search during systematic review." AMIA ... Annual Symposium proceedings. AMIA Symposium 2013: 379-387.

Gagliardi, A. R., S. Alhabib and a. t. m. o. t. G. I. N. I. W. Group (2015). "Trends in guideline implementation: a scoping systematic review." Implementation Science 10(1): 54.

Gagliardi, A. R., C. Marshall, S. Huckson, R. James and V. Moore (2015). "Developing a checklist for guideline implementation planning: review and synthesis of guideline development and implementation advice." Implementation science : IS 10: 19-19.

Gagliardi, A. R., C. Marshall, S. Huckson, R. James and V. Moore (2015). "Developing a checklist for guideline implementation planning: review and synthesis of guideline development and implementation advice." Implementation Science 10(1): 19.

GREENHALGH, T., G. ROBERT, F. MACFARLANE, P. BATE and O. KYRIAKIDOU (2004). "Diffusion of Innovations in Service Organizations: Systematic Review and Recommendations." The Milbank Quarterly 82(4): 581-629.

Greenhalgh, T., J. Wherton, C. Papoutsi, J. Lynch, G. Hughes, C. A'Court, S. Hinder, N. Fahy, R. Procter and S. Shaw (2017). "Beyond Adoption: A New Framework for Theorizing and Evaluating Nonadoption, Abandonment, and Challenges to the Scale-Up, Spread, and Sustainability of Health and Care Technologies." J Med Internet Res 19(11): e367.

Innvær, S., G. Vist, M. Trommald and A. Oxman (2002). "Health policy-makers' perceptions of their use of evidence: a systematic review." Journal of Health Services Research & Policy 7(4): 239-244.

Jewell, C. J. and L. A. Bero (2008). "“Developing Good Taste in Evidence”: Facilitators of and Hindrances to Evidence-Informed Health Policymaking in State Government." The Milbank Quarterly 86(2): 177-208.

Kastner, M., O. Bhattacharyya, L. Hayden, J. Makarski, E. Estey, L. Durocher, A. Chatterjee, L. Perrier, I. D. Graham, S. E. Straus, M. Zwarenstein and M. Brouwers (2015). "Guideline uptake is influenced by six implementability domains for creating and communicating guidelines: a realist review." Journal of Clinical Epidemiology 68(5): 498-509.

Lewis, C. C., S. Fischer, B. J. Weiner, C. Stanick, M. Kim and R. G. Martinez (2015). "Outcomes for implementation science: an enhanced systematic review of instruments using evidence-based rating criteria." Implementation Science 10(1): 155.

Malmivaara, A. (2019). "Generalizability of findings from randomized controlled trials is limited in the leading general medical journals." Journal of Clinical Epidemiology 107: 36-41.

Montgomery, P., K. Underhill, F. Gardner, D. Operario and E. Mayo-Wilson (2013). "The Oxford Implementation Index: a new tool for incorporating implementation data into systematic reviews and meta-analyses." Journal of Clinical Epidemiology 66(8): 874-882.

National Health and Medical Research Council (NHMRC) "Guidelines for Guidelines: Implementability.".

Proctor, E., H. Silmere, R. Raghavan, P. Hovmand, G. Aarons, A. Bunger, R. Griffey and M. Hensley (2011). "Outcomes for Implementation Research: Conceptual Distinctions, Measurement Challenges, and Research Agenda." Administration and Policy in Mental Health and Mental Health Services Research 38(2): 65-76.

Shiffman, R. N., J. Dixon, C. Brandt, A. Essaihi, A. Hsiao, G. Michel and R. O'Connell (2005). "The GuideLine Implementability Appraisal (GLIA): development of an instrument to identify obstacles to guideline implementation." BMC Medical Informatics and Decision Making 5(1): 23.

Streater, A., A. Spector, E. Aguirre, J. Stansfeld and M. Orrell (2016). "ImpRess: an Implementation Readiness checklist developed using a systematic review of randomised controlled trials assessing cognitive stimulation for dementia." BMC Medical Research Methodology 16(1): 167.

Worsley, S. D., K. Oude Rengerink, E. Irving, S. Lejeune, K. Mol, S. Collier, R. H. H. Groenwold, C. Enters-Weijnen, M. Egger and T. Rhodes (2017). "Series: Pragmatic trials and real world evidence: Paper 2. Setting, sites, and investigator selection." Journal of Clinical Epidemiology 88: 14-20.

Zuidgeest, M. G. P., P. M. J. Welsing, G. J. M. W. van Thiel, A. Ciaglia, R. Alfonso-Cristancho, L. Eckert, M. J. C. Eijkemans and M. Egger (2017). "Series: Pragmatic trials and real world evidence: Paper 5. Usual care and real life comparators." Journal of Clinical Epidemiology 90: 92-98.

C. Preliminary list of relevant tools

Chan A-W, Tetzlaff JM, Altman DG, Laupacis A, Gøtzsche PC, Krleža-Jerić K, et al. SPIRIT 2013 Statement: Defining Standard Protocol Items for Clinical Trials. Annals of Internal Medicine. 2013;158(3):200-7.

Hoffmann TC, Glasziou PP, Boutron I, Milne R, Perera R, Moher D, et al. Better reporting of interventions: template for intervention description and replication (TIDieR) checklist and guide. BMJ : British Medical Journal. 2014;348:g1687.

Loudon K, Treweek S, Sullivan F, Donnan P, Thorpe KE, Zwarenstein M. The PRECIS-2 tool: designing trials that are fit for purpose. BMJ : British Medical Journal. 2015;350:h2147.

Schulz KF, Altman DG, Moher D. CONSORT 2010 Statement: updated guidelines for reporting parallel group randomised trials. BMJ. 2010;340:c332.

D. Table of excluded resources

Reference		Reason for exclusion
1.	Alvarez E, Lavis J. Implementing health systems guidance: A workbook to support the contextualization of recommendations at the national or subnational level. . Hamilton, Canada: McMaster Health Forum; 2016.	Implementation
2.	Australian Government Department of Health Therapeutic Goods Administration. Australian Clinical Trial Handbook: Guidance on conducting clinical trials in Australia using ‘unapproved’ therapeutic goods. Version 2.2. Canberra: Commonwealth of Australia; 2018.	Not relevant to implementability
3.	Balshem H, Curtis P, Joplin L, Justman RA, Rosenberg AB. Stakeholder Involvement in Improving Comparative Effectiveness Reviews: AHRQ and the Effective Health Care Program. AHRQ; 2011.	Superseded by more recent guidance from same organisation
4.	Barnett J, Vasileiou K, Djemil F, Brooks L, Young T. Understanding innovators' experiences of barriers and facilitators in implementation and diffusion of healthcare service innovations: a qualitative study. BMC Health Serv Res. 2011;11:342.	Implementation

5. Brach C, Lenfestey N, Roussel A, Amoozegar J, Sorensen A. Will It Work Here? A Decisionmaker's Guide to Adopting Innovations. Publication No. 08-0051. Rockville, MD: Agency for Healthcare Research and Quality (AHRQ); 2008.	Published before 2009
6. Brach C, Lenfestey N, Roussel A, Amoozegar J, Sorensen A. Will It Work Here? A Decisionmaker's Guide to Adopting Innovations. Publication No. 08-0051. Rockville, MD: Agency for Healthcare Research and Quality (AHRQ); 2008.	Published before 2009
7. Brennan SE, Cumpston M, Misso ML, McDonald S, Murphy MJ, Green SE. Design and formative evaluation of the Policy Liaison Initiative: a long-term knowledge translation strategy to encourage and support the use of Cochrane systematic reviews for informing health policy. Evidence & Policy: A Journal of Research, Debate and Practice. 2016;12(1):25-52.	Primary study, systematic review is available.
8. Breuer E, Lee L, De Silva M, Lund C. Using theory of change to design and evaluate public health interventions: a systematic review. Implementation Science. 2016;11(1):63.	Not relevant to implementability
9. Buffet C, Ciliska D, Thomas H. Can I Use This Evidence in my Program Decision? Assessing Applicability and Transferability of Evidence2007.	Published before 2009
10. Buffet C, Ciliska D, Thomas H. Can I Use This Evidence in my Program Decision? Assessing Applicability and Transferability of Evidence2007.	Published before 2009
11. Buffet C, Ciliska D, Thomas H. It worked there. Will it work here? Tool for Assessing Applicability and Transferability of Evidence (A: When considering starting a new program)2011.	Implementation
12. Buffet C, Ciliska D, Thomas H. It worked there. Will it work here? Tool for Assessing Applicability and Transferability of Evidence (B: When considering stopping a new program)2011.	Implementation
13. Campbell M, Katikireddi SV, Hoffmann T, Armstrong R, Waters E, Craig P, et al. TIDieR-PHP: a reporting guideline for population health and policy interventions. BMJ 2018;361 k1079.	Duplicate
14. Campbell S, Benita S, Coates E, Davies P, Penn G. Analysis for policy: evidence-based policy in practice. London: HM Treasury; 2007.	Published before 2009
15. Carey T, Sanders G, Viswanathan M ea. Framework for Considering Study Designs for Future Research Needs [Internet]. Rockville (MD): Agency for Healthcare Research and Quality (US); Mar. (Methods Future Research Needs Reports, No. 8.) Available from: https://www.ncbi.nlm.nih.gov/books/NBK95273/. 2012.	Not relevant to implementability
16. CIHR. Knowledge User Engagement [Available from: http://www.cihr-irsc.gc.ca/e/49505.html	More detailed resource available from same organisation.
17. Clearly Communicating Research Results across the Clinical Trials Continuum: National Institutes of Health; 2016 [Available from: https://www.nih.gov/health-information/nih-clinical-research-trials-you/clearly-communicating-research-results-across-clinical-trials-continuum.	Not relevant to implementability
18. Cook JM, O'Donnell C, Dinnen S, Coyne JC, Ruzek JI, Schnurr PP. Measurement of a model of implementation for health care: toward a testable theory. Implementation Science. 2012;7(1):59.	Implementation
19. Damschroder LJ, Aron DC, Keith RE, Kirsh SR, Alexander JA, Lowery JC. Fostering implementation of health services research findings into practice: a consolidated framework for advancing implementation science. Implementation Science. 2009;4(1):50.	Implementation
20. de Savigny D, Adam T, editors. Systems thinking for health systems strengthening. Geneva: World Health Organization; 2009.	Implementation
21. Dechartres A, Trinquart L, Atal I, Moher D, Dickersin K, Boutron I, et al. Evolution of poor reporting and inadequate methods over time in 20 920 randomised controlled trials included in Cochrane reviews: research on research study. Bmj. 2017;357:j2490.	Duplicate

22. Dobbins M, DeCorby K, Twiddy T. A Knowledge Transfer Strategy for Public Health Decision Makers. Worldviews on Evidence-Based Nursing. 2004;1(2):120-8.	Published before 2009
23. Edinger T, Cohen AM. A large-scale analysis of the reasons given for excluding articles that are retrieved by literature search during systematic review. AMIA Annual Symposium proceedings AMIA Symposium. 2013;2013:379-87.	Not relevant to implementability
24. Ellen ME, Lavis JN, Horowitz E, Berglas R. How is the use of research evidence in health policy perceived? A comparison between the reporting of researchers and policy-makers. Health Res Policy Syst. 2018;16(1):64.	Not relevant to implementability
25. Feldstein AC, Glasgow RE. A Practical, Robust Implementation and Sustainability Model (PRISM) for Integrating Research Findings into Practice. Joint Commission Journal on Quality and Patient Safety. 2008;34(4):228-43.	Published before 2009
26. Fischer F, Lange K, Klose K, Greiner W, Kraemer A. Barriers and Strategies in Guideline Implementation-A Scoping Review. Healthcare (Basel, Switzerland). 2016;4(3).	Implementation
27. Forbes G, Loudon K, Treweek S, Taylor SJC, Eldridge S. Understanding the applicability of results from primary care trials: lessons learned from applying PRECIS-2. Journal of Clinical Epidemiology. 2017;90:119-26.	Not relevant to implementability
28. Gagliardi AR, Alhabib S, Group atmotGINIW. Trends in guideline implementation: a scoping systematic review. Implementation Science. 2015;10(1):54.	Implementation
29. Gagliardi AR, Brouwers MC, Bhattacharyya OK. The guideline implementability research and application network (GIRAnet): an international collaborative to support knowledge exchange: study protocol. Implement Sci. 2012;7:26.	Study protocol
30. Gagliardi AR, Marshall C, Huckson S, James R, Moore V. Developing a checklist for guideline implementation planning: review and synthesis of guideline development and implementation advice. Implementation Science. 2015;10(1):19.	Implementation
31. Godwin M, Ruhland L, Casson I, MacDonald S, Delva D, Birtwhistle R, et al. Pragmatic controlled clinical trials in primary care: the struggle between external and internal validity. BMC Med Res Methodol. 2003;3:28.	Published before 2009
32. Gollust SE, Seymour JW, Pany MJ, Goss A, Meisel ZF, Grande D. Mutual Distrust: Perspectives From Researchers and Policy Makers on the Research to Policy Gap in 2013 and Recommendations for the Future. Inquiry : a journal of medical care organization, provision and financing. 2017;54:46958017705465.	Primary study, systematic review is available.
33. Greenhalgh T, Robert G, Macfarlane F, Bate P, Kyriakidou O. Diffusion of Innovations in Service Organizations: Systematic Review and Recommendations. The Milbank Quarterly. 2004;82(4):581-629.	Published before 2009
34. Greenhalgh T, Wherton J, Papoutsi C, Lynch J, Hughes G, A'Court C, et al. Analysing the role of complexity in explaining the fortunes of technology programmes: empirical application of the NASSS framework. BMC Medicine. 2018;16(1):66.	Not relevant to clinical trials
35. Greenhalgh T, Wherton J, Papoutsi C, Lynch J, Hughes G, A'Court C, et al. Beyond Adoption: A New Framework for Theorizing and Evaluating Nonadoption, Abandonment, and Challenges to the Scale-Up, Spread, and Sustainability of Health and Care Technologies. J Med Internet Res. 2017;19(11):e367.	Implementation
36. Gupta A, Thorpe C, Bhattacharyya O, Zwarenstein M. Promoting development and uptake of health innovations: The Nose to Tail Tool [version 1; peer review: 3 approved, 1 approved with reservations]. (https://doi.org/10.12688/f1000research.8145.1). F1000Research. 2016;5:361.	Implementation

37.	Haynes B. Can it work? Does it work? Is it worth it? The testing of healthcare interventions is evolving. 1999;319(7211):652-3.	Published before 2009
38.	Haynes B, Haines A. Barriers and bridges to evidence based clinical practice. BMJ. 1998;317(7153):273-6.	Published before 2009
39.	He J, Du L, Liu G, Fu J, He X, Yu J, et al. Quality assessment of reporting of randomization, allocation concealment, and blinding in traditional Chinese medicine RCTs: a review of 3159 RCTs identified from 260 systematic reviews. Trials. 2011;12:122.	Specific clinical area, broader resource is available.
40.	Hyder AA, Corluka A, Winch PJ, El-Shinnawy A, Ghassany H, Malekafzali H, et al. National policy-makers speak out: are researchers giving them what they need? Health policy and planning. 2011;26(1):73-82.	Primary study, systematic review is available.
41.	Innvær S, Vist G, Trommald M, Oxman A. Health policy-makers' perceptions of their use of evidence: a systematic review. Journal of Health Services Research & Policy. 2002;7(4):239-44.	Published before 2009
42.	Jewell CJ, Bero LA. “Developing Good Taste in Evidence”: Facilitators of and Hindrances to Evidence-Informed Health Policymaking in State Government. The Milbank Quarterly. 2008;86(2):177-208.	Published before 2009
43.	Kalkman S, van Thiel GJ, Grobbee DE, van Delden JJ. Pragmatic randomized trials in drug development pose new ethical questions: a systematic review. Drug discovery today. 2015;20(7):856-62.	Not relevant to implementability
44.	L&M Policy Research, Quintiles Outcome. Registry of Patient Registries Outcome Measures Framework: Literature Review Findings and Implications. OMF Literature Review Report. (Prepared under Contract No. 290-2014-00004-C.) AHRQ Publication No. 16-EHC036-EF. Rockville, MD: Agency for Healthcare Research and Quality; 2016. Available from: www.effectivehealthcare.ahrq.gov/reports/final/cfm.	Not relevant to clinical trials
45.	Lavis J, Davies H, Oxman A, Denis J-L, Golden-Biddle K, Ferlie E. Towards systematic reviews that inform health care management and policy-making. Journal of Health Services Research & Policy. 2005;10(1_suppl):35-48.	Published before 2009
46.	LAVIS JN, ROBERTSON D, WOODSIDE JM, McLEOD CB, ABELSON J. How Can Research Organizations More Effectively Transfer Research Knowledge to Decision Makers? The Milbank Quarterly. 2003;81(2):221-48.	Published before 2009
47.	Lavis JN, Ross SE, Hurley JE. Examining the Role of Health Services Research in Public Policymaking. The Milbank Quarterly. 2002;80(1):125-54.	Published before 2009
48.	Leavy M, Schur C, Kassamali F, Johnson M, Sabharwal R, Wallace P, et al. Development of Harmonized Outcome Measures for Use in Patient Registries and Clinical Practice: Methods and Lessons Learned. Final Report. Rockville MD: Agency for Healthcare Research and Quality; 2019. Report No.: AHRQ Publication No. 19-EHC008-EF.	Not relevant to clinical trials
49.	Lewis CC, Fischer S, Weiner BJ, Stanick C, Kim M, Martinez RG. Outcomes for implementation science: an enhanced systematic review of instruments using evidence-based rating criteria. Implementation Science. 2015;10(1):155.	Implementation
50.	Li T, Mayo-Wilson E, Fusco N, Hong H, Dickersin K. Caveat emptor: the combined effects of multiplicity and selective reporting. Trials. 2018;19(1):497.	Not relevant to implementability
51.	Lomas J, Fulop N, Gagnon D, Allen P. On Being a Good Listener: Setting Priorities for Applied Health Services Research. The Milbank Quarterly. 2003;81(3):363-88.	Priority setting.
52.	Lu Y, Yao Q, Gu J, Shen C. Methodological reporting of randomized clinical trials in respiratory research in 2010. Respiratory care. 2013;58(9):1546-51.	Specific clinical area, broader resource is available.

53. Madurasinghe VW, Group SEoboMS, Group GFobotSEC. Guidelines for reporting embedded recruitment trials. Trials. 2016;17(1):27.	Not relevant to implementability
54. Morestin F. Knowledge Sharing and Public Policies: A Representation of Influence Processes2015.	Implementation
55. National Institute for Health and Care Excellence (NICE). Multimorbidity: clinical assessment and management. NICE; 2016.	Not relevant to clinical trials
56. Nieuwenhuis JB, Irving E, Oude Rengerink K, Lloyd E, Goetz I, Grobbee DE, et al. Pragmatic trial design elements showed a different impact on trial interpretation and feasibility than explanatory elements. J Clin Epidemiol. 2016;77:95-100.	Case study
57. Nilsen P, Bernhardsson S. Context matters in implementation science: a scoping review of determinant frameworks that describe contextual determinants for implementation outcomes. BMC Health Services Research. 2019;19(1):189.	Implementation
58. Orton L, Lloyd-Williams F, Taylor-Robinson D, O'Flaherty M, Capewell S. The use of research evidence in public health decision making processes: systematic review. PLoS One. 2011;6(7):e21704.	More recent systematic review available.
59. O'Sullivan AK, Thompson D, Drummond MF. Collection of health-economic data alongside clinical trials: is there a future for piggyback evaluations? Value in health : the journal of the International Society for Pharmacoeconomics and Outcomes Research. 2005;8(1):67-79.	Published before 2009
60. Otten JJ, Dodson EA, Fleischhacker S, Siddiqi S, Quinn EL. Getting research to the policy table: a qualitative study with public health researchers on engaging with policy makers. Preventing chronic disease. 2015;12:E56.	Not relevant to implementability
61. Page MJ, McKenzie JE, Kirkham J, Dwan K, Kramer S, Green S, et al. Bias due to selective inclusion and reporting of outcomes and analyses in systematic reviews of randomised trials of healthcare interventions. The Cochrane database of systematic reviews. 2014(10):Mr000035.	Not relevant to clinical trials
62. PCORI. PCORI's Stakeholders 2018 [Available from: https://www.pcori.org/about-us/our-programs/engagement/public-and-patient-engagement/pcoris-stakeholders.	Not relevant to implementability
63. PCORI (Patient Centered Ouctomes Research Institute). PCORI Dissemination and Implementation Framework. Washington, DC: PCORI; 2015.	Implementation
64. PCORI (Patient Centered Ouctomes Research Institute). PCORI Dissemination and Implementation Toolkit. Washington, DC: PCORI; 2015.	Implementation
65. Phillips AC, Lewis LK, McEvoy MP, et al. Development and validation of the guideline for reporting evidence-based practice educational interventions and teaching (GREET). BMC Med Educ. 2016;16.	Not relevant to clinical trials
66. Piaggio G, Elbourne DR, Pocock SJ, Evans SJW, Altman DG, CONSORT Group ft. Reporting of Noninferiority and Equivalence Randomized Trials: Extension of the CONSORT 2010 StatementReporting Noninferiority and Equivalence. JAMA. 2012;308(24):2594-604.	Not relevant to implementability
67. Preston NJ, Farquhar MC, Walshe CE, Stevinson C, Ewing G, Calman LA, et al. Strategies designed to help healthcare professionals to recruit participants to research studies. Cochrane Database of Systematic Reviews. 2016(2).	Not relevant to implementability
68. Rendell JM, Merritt RK, Geddes J. Incentives and disincentives to participation by clinicians in randomised controlled trials. Cochrane Database of Systematic Reviews. 2007(2).	Published before 2009
69. Rendell JM, Merritt RK, Geddes J. Incentives and disincentives to participation by clinicians in randomised controlled trials. Cochrane Database of Systematic Reviews. 2007(2).	Published before 2009
70. ROB-ME tool	In progress

71.	Rojas Smith L, Ashok M, Dy S, Wines R, Teixeira-Poit S. Contextual Frameworks for Research on the Implementation of Complex System Interventions. Methods Research Report. (Prepared by the RTI International– University of North Carolina at Chapel Hill Evidence-based Practice Center under Contract No. 290-2007-10056-I.) AHRQ Publication No. 14-EHC014-EF. Rockville, MD: Agency for Healthcare Research and Quality; 2014. Available from: www.effectivehealthcare.ahrq.gov/reports/final.cfm.	Implementation
72.	Saltaji H, Armijo-Olivo S, Cummings GG, Amin M, Flores-Mir C. Randomized clinical trials in dentistry: Risks of bias, risks of random errors, reporting quality, and methodologic quality over the years 1955-2013. PLoS One. 2017;12(12):e0190089.	Specific clinical area, broader resource is available.
73.	Scott SD, Albrecht L, O'Leary K, Ball GD, Hartling L, Hofmeyer A, et al. Systematic review of knowledge translation strategies in the allied health professions. Implementation Science. 2012;7(1):70.	Implementation
74.	Shiffman RN, Dixon J, Brandt C, Essaihi A, Hsiao A, Michel G, et al. The GuideLine Implementability Appraisal (GLIA): development of an instrument to identify obstacles to guideline implementation. BMC Medical Informatics and Decision Making. 2005;5(1).	Published before 2009
75.	Staley K. 'Is it worth doing?' Measuring the impact of patient and public involvement in research. Research Involvement and Engagement. 2015;1(1):6.	Not relevant to clinical trials
76.	Staniszewska S, Brett J, Simera I, Seers K, Mockford C, Goodlad S, et al. GRIPP2 reporting checklists: tools to improve reporting of patient and public involvement in research. BMJ. 2017;358:j3453.	Not relevant to clinical trials
77.	To MJ, Jones J, Emara M, Jadad AR. Are reports of randomized controlled trials improving over time? A systematic review of 284 articles published in high-impact general and specialized medical journals. PLoS One. 2013;8(12):e84779.	Specific clinical area, broader resource is available.
78.	Treweek S, Pitkethly M, Cook J, Fraser C, Mitchell E, Sullivan F, et al. Strategies to improve recruitment to randomised trials. Cochrane Database of Systematic Reviews. 2018(2).	Not relevant to implementability
79.	Tricco AC, Cardoso R, Thomas SM, Motiwala S, Sullivan S, Kealey MR, et al. Barriers and facilitators to uptake of systematic reviews by policy makers and health care managers: a scoping review. Implementation Science. 2016;11(1):4.	Not relevant to clinical trials
80.	Tricco AC, Cardoso R, Thomas SM, Motiwala S, Sullivan S, Kealey MR, et al. Barriers and facilitators to uptake of systematic reviews by policy makers and health care managers: a scoping review. Implement Sci. 2016;11:4.	Duplicate
81.	Turner L, Shamseer L, Altman DG, Weeks L, Peters J, Kober T, et al. Consolidated standards of reporting trials (CONSORT) and the completeness of reporting of randomised controlled trials (RCTs) published in medical journals. The Cochrane database of systematic reviews. 2012;11:Mr000030.	Duplicate
82.	Van Spall HG, Toren A, Kiss A, Fowler RA. Eligibility criteria of randomized controlled trials published in high-impact general medical journals: a systematic sampling review. Jama. 2007;297(11):1233-40.	Published before 2009
83.	Vasileiou K, Barnett J, Young T. The production and use of evidence in health care service innovation: a qualitative study. Evaluation & the health professions. 2013;36(1):93-105.	Primary study, systematic review is available.

84. Viswanathan M, Cook Middleton J, Forman-Hoffman V. Supplemental Project to Assess the Transparency of Reporting for Strategies to Improve Mental Health Care for Children and Adolescents. Methods Research Report. (Prepared by the RTI International–University of North Carolina Evidence-based Practice Center under Contract No. 290-2015-00011-I and 290-32004-T . AHRQ Publication No.17-EHC002-EF. Rockville, MD: Agency for Healthcare Research and Quality.; 2017.	Specific clinical area, broader resource is available.
85. Wallace J, Byrne C, Clarke M. Making evidence more wanted: a systematic review of facilitators to enhance the uptake of evidence from systematic reviews and meta-analyses. International journal of evidence-based healthcare. 2012;10(4):338-46.	Not relevant to clinical trials
86. Wallace J, Nwosu B, Clarke M. Barriers to the uptake of evidence from systematic reviews and meta-analyses: a systematic review of decision makers' perceptions. BMJ Open. 2012;2(5).	Not relevant to clinical trials
87. White C, Sanders Schmidler G, Borsky A, Butler M, Wang Z, Robinson K, et al. Understanding Health-Systems' Use of and Need for Evidence To Inform Decisionmaking. Research White Paper. (Prepared by the University of Connecticut and Duke Evidence-based Practice Centers under Contract No. 290-2015-00012-I and 290-2015-00004-I.) AHRQ Publication No. 17(18)-EHC035-EF. Rockville, MD: Agency for Healthcare Research and Quality; 2017.	Primary study, systematic review is available.
88. Woolacott N, Corbett M, Jones-Diette J, Hodgson R. Methodological challenges for the evaluation of clinical effectiveness in the context of accelerated regulatory approval: an overview. Journal of Clinical Epidemiology. 2017;90:108-18.	Not relevant to implementability
89. Zwarenstein M, Treweek S, Gagnier JJ, Altman DG, Tunis S, Haynes B, et al. Improving the reporting of pragmatic trials: an extension of the CONSORT statement. BMJ. 2008;337:a2390.	Published before 2009
