# Supplementary material for: Understanding implementability in clinical trials: a pragmatic review and concept map
Source: Trials. 2021 Mar 26;22:232. doi: 10.1186/s13063-021-05185-w (PMC7995762; doi:10.1186/s13063-021-05185-w)
Supplement: Supplementary file 4 — Additional file 4: Figure. Supporting resources for each concept. A more detailed version of the concept map displaying the specific supporting references for each concept. [file 13063_2021_5185_MOESM4_ESM.pdf]

Additional File 4: Figure: Supporting resources for each concept

Understanding implementability in clinical trials: a pragmatic review and concept map

Miranda Cumpston, Steve Webb, Philippa Middleton, Greg Sharplin, Sally Green for the Australian Clinical Trials Alliance Reference Group on Impact and Implementation of CTN Trials.

	Design	Resources*	Conduct	Resources*	Reporting	Resources*
Validity	Trial protocol complete	14 12-3	All randomised participants included in ITT analysis	63,67 40	Reporting complete	14,26 28,62 5,56-7
	Team has methodology training & includes a statistician	34			*Sufficient information to assess risk of bias*	14,26,34,46 48 3,18,21,28,53
	Protocol uses standardised statistical/analytical procedures	34				29,36
					No selective reporting	14,26 22,30,35,37,54,66
					Declaration of interests	34,46 48
Relevance	*Design informed by stakeholdes & end users*	4,9,16,17,20,33,45,46,63,65	Analysis & conclusions meaningful to stakeholders	17	*Sufficient information to assess applicability*	1,14,25,26,39,45,52,61 28,41
		23,24,59 48 50				2,7-8,27,36,43,55,60
	Pragmatic design	34			Information relevant to different stakeholders	45
	**Population**				Rationale for selection/exclusion of harm outcomes	15
	Realistic (inclusive of diverse populations & risks)	46,49,61,63,67 40,64				
	Feasible diagnostic/eligibility criteria.	40				
	Actively seeking diversity (for applicability, risk stratification)	49				
	Sites comparable to where implementation occurs	61,65 40,64				
	**Interventions & comparators**					
	Feasible & acceptable in current practice & systems	16,46,51,52 8,36				
	Intervention as it would be delivered in practice	49,61,67 20,64				
	Comparators based in current practice	9,67 7-8,64				
	Evidence of current clinical practice is available	46,67				
	**Outcomes**					
	Important to end users	9,15,16,17,25,34,38,45,61,63				
		7-8,19,40				
	Routine collection	63,65 40				
	Pragmatic collection	63				
Usability	Registered	32,34,47	Procedures, training & other materials retained	25	Published, regardless of outcome	14,32,47 22,48,68
	Protocol published	34,47		31,42,60	Accessible to end users	14,32 48
	Specify theory/logic model of how intervention will work	45			Clearly reported	48
	**Outcomes**				*Sufficient information to replicate*	25,26,34,45,46 44
	Consent obtained for data re-use	58				10,11,27,31,36,42,43
	Fidelity measures included	6,45,51,52 11,31,42			Authors responsive to requests for data	14,34,47
	Process information/evaluation/feasibility included	25,45 11,42,43,60				
	Economic information/evaluation included	9,25,38,45,46,52 7-8,42,60				

*Resources refer to the number listed in the Characteristics of Included Resources table (Additional File 3). Resource types: Guidance, Descriptive data (benefit), Descriptive data (problem), Tool.

Concepts in *italics* have ≥10 supporting resources.
